# Nicotine Synergizes with High-Fat Diet to Induce an Anti-Inflammatory Microenvironment to Promote Breast Tumor Growth

**DOI:** 10.1155/2020/5239419

**Published:** 2020-12-10

**Authors:** Thalia Jimenez, Theodore Friedman, Jaydutt Vadgama, Vineeta Singh, Alexandria Tucker, Javier Collazo, Satyesh Sinha, Amiya Sinha Hikim, Rajan Singh, Shehla Pervin

**Affiliations:** ^1^Division of Endocrinology and Metabolism, Department of Medicine, Charles R. Drew University of Medicine and Science, Los Angeles, CA 90059, USA; ^2^Department of Medicine, David Geffen School of Medicine at UCLA, Los Angeles, CA 90095, USA; ^3^Jonsson Comprehensive Cancer Center, David Geffen School of Medicine at UCLA, Los Angeles, CA 90095, USA; ^4^Division of Cancer Research and Training, Department of Medicine, Charles R. Drew University of Medicine and Science, Los Angeles, CA 90059, USA; ^5^Department of Obstetrics and Gynecology, David Geffen School of Medicine at UCLA, Los Angeles, CA 90095, USA

## Abstract

Breast cancer results from a complex interplay of genetics and environment that alters immune and inflammatory systems to promote tumorigenesis. Obesity and cigarette smoking are well-known risk factors associated breast cancer development. Nicotine known to decrease inflammatory signals also modulates immune responses that favor breast cancer development. However, the mechanisms by which nicotine and obesity contribute to breast cancer remain poorly understood. In this study, we examined potential mechanisms by which nicotine (NIC) and high-fat diet (HFD) promote growth of HCC70 and HCC1806 xenografts from African American (AA) triple negative (TN) breast cancer cells. Immunodeficient mice fed on HFD and treated with NIC generated larger HCC70 and HCC1806 tumors when compared to NIC or HFD alone. Increased xenograft growth in the presence of NIC and HFD was accompanied by higher levels of tissue-resident macrophage markers and anti-inflammatory cytokines including IL4, IL13, and IL10. We further validated the involvement of these players by *in vitro* and *ex vivo* experiments. We found a proinflammatory milieu with increased expression of IL6 and IL12 in xenografts with HFD. In addition, nicotine or nicotine plus HFD increased a subset of mammary cancer stem cells (MCSCs) and key adipose browning markers CD137 and TMEM26. Interestingly, there was upregulation of stress-induced pp38 MAPK and pERK1/2 in xenografts exposed to HFD alone or nicotine plus HFD. Scratch-wound assay showed marked reduction in proliferation/migration of nicotine and palmitate-treated breast cancer cells with mecamylamine (MEC), a nicotine acetylcholine receptor (nAchR) antagonist. Furthermore, xenograft development in immune-deficient mice, fed HFD plus nicotine, was reduced upon cotreatment with MEC and SB 203580, a pp38MAPK inhibitor. Our study demonstrates the presence of nicotine and HFD in facilitating an anti-inflammatory tumor microenvironment that influences breast tumor growth. This study also shows potential efficacy of combination therapy in obese breast cancer patients who smoke.

## 1. Introduction

Molecular and cellular alterations influenced by genetic as well as environmental factors contribute to breast cancer development [1]. Poor understanding of the complexity of breast cancer has resulted in ineffective treatment strategies and high mortality rate. In addition, factors that increase the risk for breast cancer development also remain understudied. A diverse array of engineered models including single cell sequencing has revealed highly plastic nature of breast cancer cells, which generate heterogeneous dynamic clones that change over time [2, 3]. In addition, these clones cross-talk with complex breast tumor microenvironment, which actively not only participate in tumor development but also manipulate therapeutic responses [4, 5]. Despite these challenges, prognosis for breast cancer types that present estrogen receptor (ER), progesterone receptor (PR), and/or HER2/neu has improved due to targeted drug development. However, specific pharmaceutical countermeasures have not been successful in treating breast cancers that are negative for ER, PR, and Her2 neu, called triple-negative breast cancers (TNBC). However, 330,000 American women develop breast cancer and 41,760 die of the disease per year; thus, research needs to focus on prevention that will require a deeper understanding of the environmental factors that influence breast tumor development.

Both active and passive smoking has been shown to be a major risk factor for the initiation and progression of breast cancer [6–8]. Burning of the 600 ingredients in cigarettes produces more than 4,000 chemicals of which 60 are known carcinogens [9]. Nicotine, the main addictive ingredient in cigarettes, is also present in nicotine-replacement therapy (NRT), which is available to many smokers who want to stop smoking. Furthermore, since nicotine cessation leads to weight gain, especially in women, who have stopped smoking and continue on NRT to prevent weight gain [10], the consequences of nicotine from cigarettes versus from NRT on breast cancer development are difficult to delineate in humans, thus emphasizing the importance of animal models. Nicotine, a tumor-promoting compound, binds to the nicotinic acetylcholine receptors (nAchR) to promote tumor growth by increasing angiogenesis and metastasis [11]. Breast tumors with higher levels of nAchR are usually more aggressive and have poor prognosis [12]. Nicotine influences the development of various subtypes of breast tumors through PI3K/Akt/Ras/Raf and STAT1/STAT3 pathways [13, 14]. However, more alarming is the fact that nicotine could contribute to breast tumor metastasis by increasing the expression of a GTPase, cdc42, as well as vimentin, which promotes epithelial to mesenchymal transition (EMT), a key step in metastasis [15]. Another mechanism by which nicotine promotes breast tumor development is by inducing galectin-3, an antiapoptotic *β*-galactoside-binding lectin that binds *α*9-nAchR to activate the STAT signaling pathway [16].

Interestingly, there are reports of nicotine increasing aldehyde dehydrogenase 1- (ALDH1-) expressing mammary cancer stem cell population (MCSC) in MCF7 breast cancer cell line [15]. Mechanistically, nicotine increased Hes-1 expression, a target gene involved in the Notch signaling pathway to facilitate stem cell renewal [17]. MCSCs are a small stem cell population in breast tumors that have the potential to promote initiation and progression of breast tumors [18]. Heterogeneous populations of MCSCs express markers like ALDH1, CD44, and/or CD133, which has enabled their identification [19]. Alarmingly, there are high levels of ALDH1 expression in aggressive Ghanaian and African American (AA) TNBC [20]. Although a large population of AA women who develop breast tumors also smoke, the influence of nicotine-induced various cytokines on MCSCs and tumor growth remains understudied.

Obesity is a known risk factor for breast cancer [21, 22]. Breast tumors develop in an adipose-rich microenvironment, which has proven to be an active participant in the tumor development [23]. Cytokines, hormones, and growth factors secreted from adipocytes can induce phenotypic changes and aggressive behavior in breast cancer cells [24]. A cross-talk between cancer cells and adipocytes in tumor microenvironment generate Cancer-Associated Adipocytes (CAA) that undergo morphological changes and acquire additional secretory capability to influence breast tumor development [25]. Oncostatin M (OSM), a paracrine secretion from CAA, increased STAT3 phosphorylation subsequently inducing angiogenesis in breast cancer xenografts [26]. Conditioned media from CAA were sufficient to induce migration of MCF-7, demonstrating potency of secreted cytokines [27]. Obese people have high circulating cytokines as well as pockets of cytokine secreting cells that attract immune cells, such as macrophages, resulting in sustained inflammatory sites that could be the fertile ground for cancer development [28]. However, breast tumors are heterogeneous where pockets of both M1 macrophages and proinflammatory cytokines (TNF*α*, IL-12, and IL-6) as well as M2 macrophages and anti-inflammatory cytokines exist [28]. Despite implications of both M1 and M2 macrophages in breast cancer progression, M2 macrophages with its wound healing properties promote angiogenesis and metastasis in aggressive breast tumors [29]. M2 macrophages have also been reported to cross-talk with beige/brown adipocytes, which are upregulated by nicotine. We have recently demonstrated an increase in beige adipocytes in xenografts from breast cancer cells and patient tumors, where it contributes to tumor growth [30].

Recent studies suggest that obese AA women are more susceptible to develop an aggressive form of TNBC for which there is no treatment [31]. In addition, a large number of AA women who are obese and develop aggressive form of breast cancer also smoke [32]. Mechanisms by which smoking contributes to the development of this aggressive disease in obese AA women remains poorly understood. In this study, we used immune-deficient nude mice to examine mechanisms by which nicotine (NIC) and high-fat diet (HFD) promote growth of xenografts from AA TN breast cancer cells. We examined whether nicotine promotes conversion of HFD-induced increased adipose tissue to beige adipocytes, as well as in-filtered M1 macrophages to tumor promoting M2 phenotype.

## 2. Materials and Methods

### 2.1. Cell Culture

Human breast cancer cell lines HCC1806 and HCC70 were purchased from American Type Culture Collection (ATCC) (Manassas, VA). Cells were maintained in RPMI 1640 (Thermo Fisher Scientific, Pittsburgh, PA) containing 10% fetal bovine serum (FBS) (ATCC, Manassas, VA). Cell line authentication was done at ATCC, which uses Promega PowerPlex 1.2 system and the Applied Biosystems Genotype 2.0 software for analysis of amplicon.

### 2.2. Xenograft Formation

Immunodeficient mice (6-8 weeks old from Envigo, cat# 6903F) were fed normal or HFD (60% kcal from fat) (Research Diets, Inc., cat # D12492) for two weeks. Simultaneously, control and HFD received intraperitoneal (IP) injections of nicotine (0.75 mg/kg/mice) twice a day. After two weeks of pretreatment, breast cancer cell line HCC70 (2×10^6^ cell/100 *μ*l) mixed with Matrigel (1 : 1) (Corning, Manassas, VA) was injected subcutaneously in nude mice for xenograft growth. Xenografts were allowed to grow for another 8 weeks in mice continued on HFD with twice daily injections of nicotine during which the growth of xenografts was monitored weekly until mice were euthanized and tumors excised. Mice, 5/group exposed to only saline, HFD, or nicotine were used as appropriate controls. In another experiment, HFD- and NIC-pretreated mice, injected with breast cancer cells, were treated with intraperitoneal injection of mecamylamine (MEC) (Sigma CAS#826-39-1) (0.75 mg/kg/mice, twice a day/every day) as well as subcutaneous injection of SB 203580 (Calbiochem, San Diego, cat# CAS 869185-85-3) (0.2 *μ*mols in 100 *μ*l/per 20 g mice once daily; every day). We monitored xenograft growth (5 mice/group), weekly for 10 weeks following which the mice were euthanized and xenografts excised. Xenografts were examined for the expression of various inflammatory and stem cell markers by quantitative real-time PCR and immunoblot analysis or immunohistochemical staining [33].

### 2.3. Immunoblot Analysis

Xenografts were homogenized with T-PER reagent (Thermo Fisher Scientific, Rockford, IL), and protein concentrations were determined using Pierce BCA Protein assay kit (Thermo Fisher Scientific, cat# 88667) and Spectra Max spectrophotometer (Model Spectra Max 190) at 545 nm. 50-100 *μ*g of cell or tissue lysates were resolved on 10%-15% SDS-PAGE gels, and electrotransferred to polyvinylidene difluoride (PVDF) membranes (Bio-Rad, Hercules, CA, cat# 1620177). The membranes were incubated with the following primary antibodies at 1 : 1,000 dilutions: ERK1/2 (Cell Signaling Technology, cat# 9102), pERK1/2 (Cell Signaling Technology, cat# 9106), ALDH1 (Abcam, ab9883), OCT4 (Stem Cell Technologies, cat# 60059), SOX2 (Abcam, ab97959), p38MAPK (Cell Signaling Technology, cat# 9212), pp38 MAPK (Cell Signaling Technology, cat# 9211), CD68 (Abcam, ab76308), CD163 (Abcam, ab182422), IL13 (Abcam, ab9576), IL12 (Santa Cruz BioTech, sc74147), IL10 (Santa Cruz BioTech, sc8438), IL6 (Santa Cruz Biotech, sc80111), MCSF (Santa Cruz BioTech, sc365779), TMEM26 (Novus Biologicals, NBP2 27334), VEGF (Abcam, ab46154), vimentin (Hybridoma Bank, AMF17b-s), and *β*-actin (Santa Cruz BioTech, sc81178). After 2 h incubation with the primary antibodies, the membranes were washed and incubated with either rabbit (Cell Signaling Technology, cat# 7074) or mouse (Cell Signaling Technology, cat# 7076) horseradish peroxidase-linked F (ab) fragment secondary antibodies (1 : 1000) for 1 h. Immunoreactive bands were visualized by enhanced chemiluminescence (ECL) detection system (Amersham, Pittsburgh, PA) as described previously [34].

### 2.4. Quantitative Real-Time PCR

Total RNA from various xenografts were extracted by Trizol reagent (Thermo Fisher Scientific), and 2 *μ*g of total RNA was reversely transcribed to cDNA using RNA High Capacity cDNA kit (Applied Biosystems, Foster City, CA, USA). Quantitative real-time PCR was conducted using fast SYBR Green PCR master mix (Thermo Fisher Scientific) and 7500 fast real-time PCR system (Applied Biosystems) [35]. Human and mouse PCR primer sequences were obtained from Primer Bank DNA Core facility (https://pga.mph.harvard.edu/primerbank/, MGH Harvard, Cambridge, MA). Primer sequences are listed in Table 1.

### 2.5. Aldefluor Assay and Flow Cytometry

Aldefluor assay was carried out as described previously [35] according to the manufacturer's (cat# 01700, Stem cell Technologies, Vancouver, Canada) guidelines. Briefly, cells suspended in Aldefluor assay buffer were incubated with 1.5 *μ*M bodipy-aminoacetaldehyde (BAAA) (ALDH substrate) for 40 min at 37°C. A fraction of the cells with BAAA was incubated with 10-fold molar excess of diethyl amino benzaldehyde DEAB, which is an ALDH inhibitor, under identical conditions and was used as a control. ALDH^+^ and ALDH^−^ cells were analyzed in a BD-LSR II analyzer (UCLA core lab). OCT4 and SOX2 expressing cells were quantitated by suspension in PBS followed by incubation with PE conjugated anti-OCT4 (cat# 3A2A20, Bio Legend, San Diego) or PE-conjugated anti-SOX2 antibodies (cat# 60447, Cell Signaling Technology, Inc., Boston) for 2 hours and subjected to flow cytometry analysis (FACSCalibur analyzer, UCLA core lab).

### 2.6. Mammosphere Formation

HCC70 cells after various treatments (NIC and ±PAL, 48 h) were suspended in mammosphere media (DMEM supplemented with 10*μ*g/ml insulin and 25 ng/ml fibroblast growth factor) and plated on ultralow attachment 6-well plates (Costar™ 3471), at a density of 50,000 cells/well as described previously [36]. We harvested the spheres at 48 h and number of mammospheres counted using an Olympus BX43 motorized microscope.

### 2.7. Immunohistochemical Analyses

Xenografts excised from nude mice were fixed in 5% formalin overnight, after which they were dehydrated in ethanol and embedded in paraffin. Tumor sections (5-6*μ*m) were deparaffinized, and immunolabeling was performed using anti-IL13 antibody (Abcam, ab9576).

### 2.8. Statistical Analyses

Data are presented as the mean ± SD, and between-group differences were analyzed using ANOVA. If the overall ANOVA revealed significant differences, then, pair-wise comparisons between groups were performed by a Newman-Keuls multiple comparison test. *p* values < 0.05 were considered statistically significant. The experiments were repeated at least three times.

## 3. Results

### 3.1. Nicotine and HFD Increase Growth of Xenografts where MCSCs and Tissue-Resident Macrophage Population Were Upregulated

**Figure 1:**
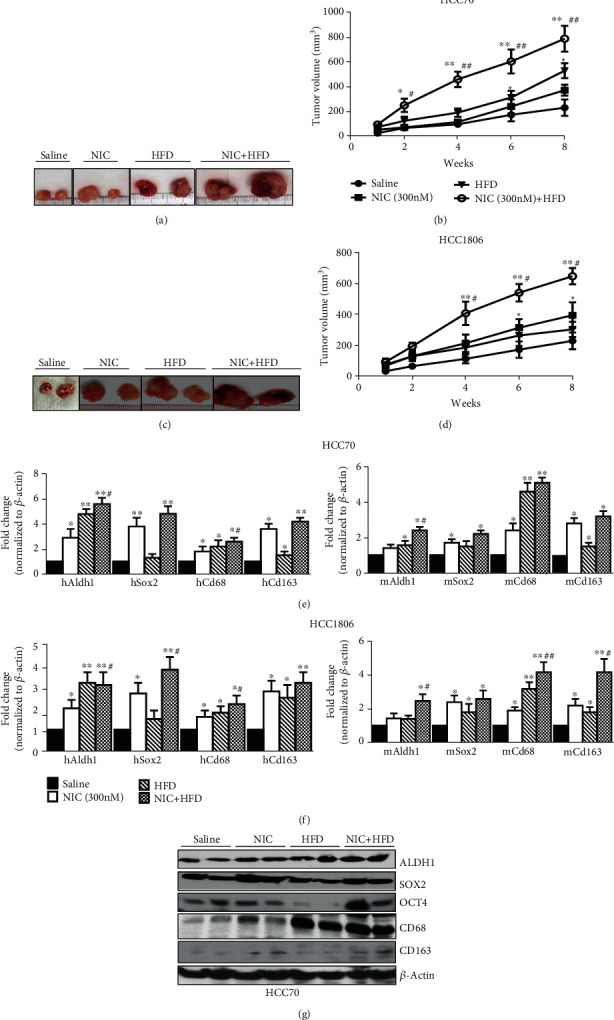
Coexposure to NIC plus HFD contributes to larger xenograft growth when compared to NIC or HFD alone. AA TN breast cancer cells HCC70 and HCC1806 (2×10^6^) were implanted subcutaneously in nude mice prefed (two weeks) HFD (rodent diet with 60 kcal % fat) and/or NIC (intraperitoneal injection twice/day, 0.75 mg/kg/mice/injection). The xenografts were allowed to grow for 8 weeks in the mice continued on HFD, NIC, or NIC plus HFD, after which the mice were euthanized and tumors excised. (a) Representative HCC70 tumors from control saline, NIC, or HFD alone or in combination (NIC+HFD) at 8 weeks (*n* = 5). (b) Weekly analysis of HCC70 tumor volume in various treatment groups. (c) Representative HCC1806 tumors from control saline, NIC, or HFD alone or in combination (NIC+HFD) at 8 weeks (*n* = 5). (d) Weekly analysis of HCC1806 tumor volume in various treatment groups. (e) Quantitative gene expression analysis of *Aldh1*, *Sox2*, *Cd68*, and *Cd163* in HCC70 xenografts from various treatment groups, using human- (left panel) and mouse- (right panel) specific primer sets. (f) Quantitative gene expression analysis of *Aldh1*, *Sox2*, *Cd68*, and *Cd163* in HCC1806 xenografts from various treatment groups, using human- (left panel) and mouse- (right panel) specific primer sets. (g) Western blot analysis of xenografts for various macrophage and mammary cancer stem cell markers. ^∗^*p* ≤ 0.05and^∗∗^*p* ≤ 0.01compared to saline or^#^*p* ≤ 0.05and^##^*p* ≤ 0.01compared to the NIC group alone (*n* = 3).

We initially examined xenograft growth in the presence of NIC and HFD from two human breast cancer cell lines HCC70 and HCC1806. Larger tumor size was observed in the NIC plus HFD group compared to the saline, NIC, or HFD groups alone with both HCC70 and HCC1806 breast cancer cell lines (Figures 1(a) and 1(c)). Further analysis of HCC70 tumor volume, over a period of 8 weeks revealed significant increase in the NIC plus HFD group (788 ± 61 mm^3^) compared to the control saline (231 ± 46 mm^3^) (*p* ≤ 0.01) or NIC (372 ± 32 mm^3^) (*p* ≤ 0.05) or HFD (529 ± 43 mm^3^) (*p* ≤ 0.01) groups alone (Figure 1(b)). Similar analysis of HCC1806 tumor volume, showed significant increase in the NIC plus HFD group (647 ± 23.6 mm^3^) compared to the control saline (229 ± 24 mm^3^) (*p* ≤ 0.01) or NIC (372 ± 32 mm^3^) (*p* ≤ 0.05) or HFD (303 ± 22 mm^3^) (*p* ≤ 0.01) groups alone (Figure 1(d)). Analysis of tumor weight for HCC70 showed significant increase in the NIC plus HFD group (738 ± 47 mg) compared to control saline (305 ± 26 mg; *p* ≤ 0.01), NIC (357 ± 24 mg; *p* ≤ 0.01), or HFD (461 ± 33 mg; *p* ≤ 0.05) groups (data not shown).

Although both HCC70 and HCC1806 xenografts showed similar increase in tumor volumes in presence of NIC + HFD, key tumorigenic factors were examined to determine whether similar mechanisms contributed to their growth. We initially examined excised tumors for key MCSCs and macrophage markers highly implicated in breast tumor development [29]. Since the host plays an important role in breast tumor development [28], we performed qPCR with species-specific primers to determine host and tumor cell contribution in MCSCs and macrophages in xenograft growth. Quantitative gene expression analysis of HCC70 xenografts showed a significant increase of MCSC markers (*Aldh1* and *Sox2*) of tumor cell (human) origin in the NIC plus HFD group, compared to the control saline group: *Aldh1* (*hAldh1*: 5.6 ± 0.5-fold, *p* ≤ 0.01; *mAldh1*: 2.4 ± 0.2-fold, *p* ≤ 0.05) and *Sox2* (*hSox2*: 4.8 ± 0.6-fold, *p* ≤ 0.01; *mSox2*: 2.2 ± 0.2-fold, *p* ≤ 0.05) (Figure 1(e)). In addition, NIC alone increased gene expression of *Sox2* (*hSox2*: 3.8 ± 0.5-fold, *p* ≤ 0.01; *mSox2*: 1.7 ± 0.2-fold, *p* ≤ 0.05) and hCd68 (1.8 ± 0.4-fold, *p* ≤ 0.05), while HFD increased *Aldh1* (*hAldh1*: 4.8 ± 0.4-fold, *p* ≤ 0.01; *mAldh1*: 1.6 ± 0.25-fold, *p* ≤ 0.05), *hCd68* (2.2 ± 0.5-fold, *p* ≤ 0.05), and *Cd163* (*hCd163*: 1.5 ± 0.3-fold, *p* ≤ 0.05; *mCd163*: 1.5 ± 0.2-fold, *p* ≤ 0.05) compared to the control saline group (Figure 1(e)). Analysis of HCC1806 xenografts also showed a similar trend in MCSC markers (*Aldh1* and *Sox2*) of tumor cell (human) origin in the NIC plus HFD group compared to the control saline group: *Aldh1* (*hAldh1*: 3.1 ± 0.6-fold, *p* ≤ 0.01; *mAldh1*: 2.5 ± 0.4-fold, *p* ≤ 0.05) and *Sox2* (*hSox2*: 3.8 ± 0.6-fold, *p* ≤ 0.01; *mSox2*: 2.6 ± 0.5-fold, *p* ≤ 0.05) (Figure 1(f)). In addition, NIC alone increased gene expression of *Sox2* (*hSox2*: 2.7 ± 0.5-fold, *p* ≤ 0.05) and *Cd68* (*hCd68*: 1.6 ± 0.3-fold, *p* ≤ 0.05; *mCd68*: 1.9 ± 0.2-fold, *p* ≤ 0.05), while HFD increased *Aldh1* (*hAldh1*: 3.2 ± 0.5-fold, *p* ≤ 0.01; *mAldh1*: 1.4 ± 0.2-fold, *p* ≤ 0.05), *hCd68* (1.8 ± 0.3-fold, *p* ≤ 0.05), and *mSox2* (1.8 ± 0.5-fold, *p* ≤ 0.05) compared to the control saline group (Figure 1(f)). We also found significantly increased expression of macrophage (*Cd68*) and tissue-resident macrophage (*Cd163*) markers in HCC70 xenografts: (*hCd68*: 2.6 ± 0.3-fold, *p* ≤ 0.05; *mCd68*: 5.1 ± 0.3-fold, *p* ≤ 0.01) and *Cd163* (*hCd163*: 4.2 ± 0.3-fold, *p* ≤ 0.01; *mCd163*: 3.2 ± 0.3-fold, *p* ≤ 0.05) in NIC + HFD group compared to the control saline group (Figure 1(e)). A similar trend was observed in HCC1806 xenograft with significantly increased expression of *Cd68*: (*hCd68*: 2.2 ± 0.4-fold, *p* ≤ 0.05; *mCd68*: 4.2 ± 0.6-fold, *p* ≤ 0.01) and *Cd163* (*hCd163*: 3.2 ± 0.5-fold, *p* ≤ 0.01; *mCd163*: 4.2 ± 0.8-fold, *p* ≤ 0.01) in the NIC + HFD group compared to the control saline group (Figure 1(f)). Since a very similar trend in the expression of key tumor promoting factors was observed in both HCC70 and HCC1806 xenografts, protein expression of these factors was further validated using HCC70 xenografts. We also examined OCT4, which in addition to SOX2 is a potent core transcription factor that governs pluripotency of embryonic stem cells. Densitometric analysis of the immunoblots after normalization with *β*-actin of HCC70 xenografts showed increased expression of key stem cell markers SOX2 (NIC: 1.4-fold; NIC + HFD: 1.46-fold), OCT4 (NIC: 1.3-fold; NIC + HFD: 1.42-fold), and ALDH1 (NIC: 1.3-fold; HFD: 1.4-fold; NIC + HFD: 1.5-fold) protein expression when compared to the control saline group (Figure 1(g)). Further analysis of protein expression of infiltrating and tissue-resident macrophage markers shows increased levels of CD68 (NIC: 1.6-fold; HFD: 1.8-fold; NIC + HFD: 1.82-fold) and CD163 (NIC: 1.4-fold; HFD: 1.28-fold; NIC + HFD: 1.94-fold) compared to their respective control saline group (Figure 1(g)). These data indicate that both independent and interdependent actions of NIC and HFD in an additive and synergistic manner may have contributed to increased xenograft growth.

### 3.2. Upregulation of Anti-Inflammatory Cytokines and MAP Kinase Signaling in Xenografts Obtained from NIC plus HFD Groups Compared to the Saline-Treated Groups

**Figure 2:**
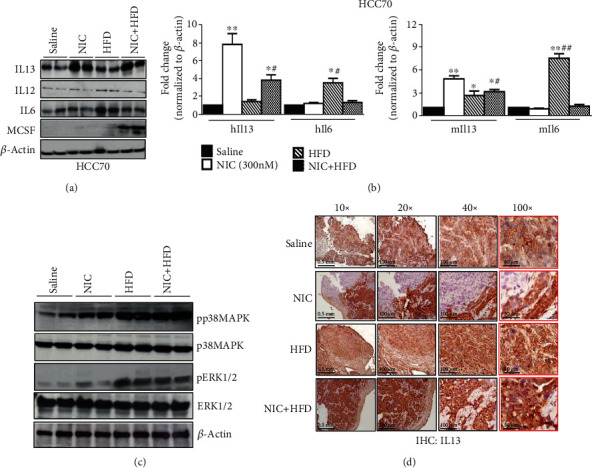
Increased expression of anti-inflammatory cytokines in xenografts exposed to either NIC or NIC+HFD. (a) Western blot analysis of cytokines IL13, IL12, IL6, and MCSF in HCC70 xenografts obtained from various treatment groups. *β*-Actin was used as control. (b) Quantitative gene expression analysis of IL13 and IL6 in HCC70 xenografts from various treatment groups, using human- (right panel) and mouse- (left panel) specific primer sets. (c) Western blot analysis of p38MAPK and pERK1/2 in xenografts obtained from various treatment groups. (d) Immunohistochemical (IHC) analysis of IL13 expression in xenografts obtained from various treatment groups. ^∗^*p* ≤ 0.05and^∗∗^*p* ≤ 0.01compared to saline or ^#^*p* ≤0.05 and ^##^*p* ≤0.01 compared to the NIC group alone (*n* = 3).

Due to similar expression pattern of macrophages in both HCC70 and HCC1806 xenografts, we further examined protein levels of proinflammatory and anti-inflammatory cytokines using HCC70 tumors. Since NIC as well as NIC + HFD xenografts contain high levels of tissue-resident macrophages (CD163), we further examined the levels of IL13, anti-inflammatory cytokine, in various treatment groups of HCC70 tumors. In addition, we also examined the levels of proinflammatory cytokines such as IL12/IL6, expressed in circulating macrophages (CD68), which also accumulate in a breast tumor microenvironment. Immunoblot analysis of HCC70 xenografts revealed significantly increased levels of anti-inflammatory cytokine IL13 (NIC: 3.9-fold; HFD: 1.2-fold; NIC + HFD: 3.1-fold) and proinflammatory cytokines IL12 (NIC: 1.3-fold; HFD: 1.6-fold) and IL6 (HFD: 1.6-fold; NIC + HFD: 1.24-fold) (Figure 2(a)). Immunoblot analysis also showed increased levels of macrophage colony-stimulating factor (MCSF), known to promote macrophage development [37], only in the NIC + HFD-treated group (5.2-fold) (Figure 2(a)). Quantitative gene expression analysis of these cytokines in various HCC70 xenograft groups using prevalidated species-specific primers show that NIC increased the expression of *hIl13* (7.8 ± 1.2-fold; *p* ≤ 0.01) and *mIl13* (4.8 ± 1.4-fold; *p* ≤ 0.01), while NIC plus HFD increased *hIl13* (3.8 ± 0.6-fold; *p* ≤ 0.05) and *mIl13* (5.1 ± 0.3-fold; *p* ≤ 0.01) (Figure 2(b)). While there was no significant change in *hIl13* expression levels in the HFD group alone compared to the saline control, *mIl13* levels were significantly upregulated (2.6 ± 0.3-fold; *p* ≤ 0.05) (Figure 2(b)). Comparison of *Il6* gene expression levels in HFD alone compared to the control saline group further showed significant increase in *Il6* (*hIl6*: 3.5 ± 0.2-fold, *p* ≤ 0.05; *mIl6*: 7.5 ± 0.3-fold; *p* ≤ 0.01) (Figure 2(b)). Since stress kinase pp38 MAPK influences HFD-induced obesity [38], we examined its levels along with that of pERK1/2 in xenografts from the NIC plus HFD treatment groups of HCC70 xenografts. Relative densitometric analyses of protein expression levels of pp38MAPK (NIC: 2.1fold; HFD: 3.7-fold; NIC + HFD: 4.2-fold) and pERK1/2 (NIC: 1.4-fold; HFD: 3.7-fold; NIC + HFD: 2.9-fold) levels when compared to saline groups alone (Figure 2(c)). Immunohistochemical analysis of paraffin-embedded sections of HCC70 xenografts from various treatment groups showed increased IL13 expression in NIC and NIC plus HFD xenografts when compared to xenografts obtained from either saline control or HFD treatment groups (Figure 2(d)). Our data indicate a role for anti-inflammatory cytokines as well as phosphorylation of p38 MAPK/ERK1/2 during NIC and NIC + HFD treatment-induced xenograft growth.

### 3.3. Nicotine and Palmitate Increase Mammary Cancer Stem Cells, Beige Markers, and Anti-Inflammatory Cytokines in Human Breast Cancer Cells *In Vitro*

**Figure 3:**
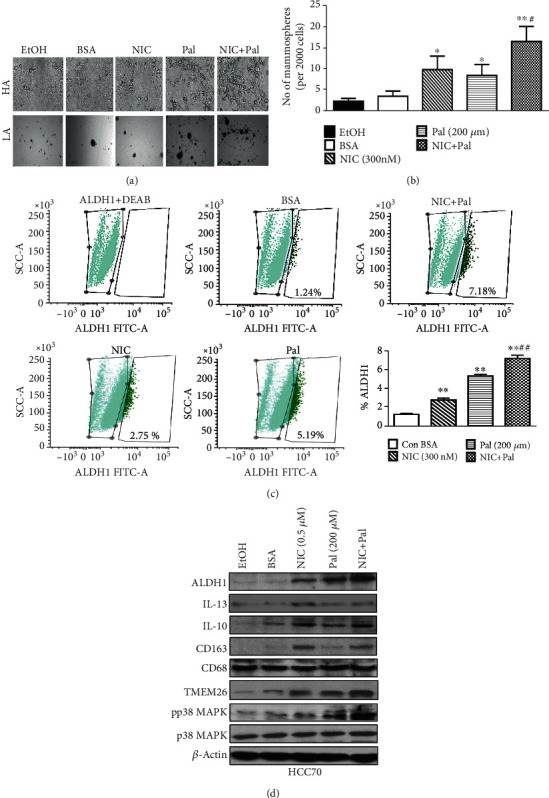
Treatment of breast cancer cells, *in vitro*, with NIC and Pal increases MCSCs, anti-inflammatory cytokines, and tissue-resident macrophage populations. (a) HCC70 cells were plated under low attachment (LA) conditions as mammospheres and treated with either NIC or BSA-conjugated palmitate (Pal) alone or in combination for 48 h. A representative picture of the mammospheres after various treatments. (b) Quantitation of the mammosphere numbers after various treatments for 48 h. (c) Treatment of HCC70 cells with NIC ± PAL or vehicles was subjected to FACS analysis after performing Aldefluor assay for quantitation of ALDH1-positive population in various treatment groups. (d) Western blot analysis of macrophage (CD68 and CD163) and anti-inflammatory (IL13 and IL10) cytokine markers, MCSCs (ALDH1), and beige adipocyte (TMEM26) marker and stress kinase (p38MAPK). ^∗^*p* ≤ 0.05and^∗∗^*p* ≤ 0.01compared to saline or^#^*p* ≤ 0.05and^##^*p* ≤ 0.01compared to the NIC group alone (*n* = 3).

Since protein expressions of MCSCs and macrophages were examined using HCC70 xenografts, we set up *in vitro* models using HCC70 cells to further validate our *in vivo* data. The effect of NIC and HFD on MCSC, macrophage populations, and anti-inflammatory cytokines was examined using *in vitro* models. Due to solubility issues of high fat in aqueous solvents, we used palmitate (Pal) conjugated with bovine serum albumin (BSA) for our *in vitro* experiments. We examined HCC70 cells treated with NIC or Pal alone or in combination for MCSCs, as well as key pro- or anti-inflammatory cytokines and macrophage markers. We enriched MCSC from HCC70 cell line by propagating under low-attachment plates as mammospheres, which are high in mammary stem cell population. There was a significant increase in the number and size of mammospheres with 300 nM NIC and 200 *μ*M Pal treatments either alone or in combination when compared to ethanol or BSA groups (Figure 3(a)). The analysis of total number of mammospheres per 50,000 cells/well from various treatment groups showed a significant increase in NIC (4.45-fold; *p* ≤ 0.05), Pal (2.5-fold; *p* ≤ 0.05), and NIC + Pal (7.5-fold; *p* ≤ 0.01) groups compared to control groups (Figure 3(b)). Quantitation of ALDH1-positive population in these groups by Aldefluor assay further shows a significant increase in NIC (2.3-fold, *p* ≤ 0.01), Pal (4.4-fold, *p* ≤ 0.001) and NIC + Pal (6.1-fold, *p* ≤ 0.001) groups compared to the control group (Figure 3(c)). Relative densitometric analyses of protein expression levels of ALDH1 (NIC: 2.4-fold; Pal: 3.3-fold; NIC + Pal: 4.2-fold), IL-13 (NIC: 1.6-fold; Pal: 1.4-fold; NIC + Pal: 1.5-fold), and IL-10 (NIC: 2.3-fold; Pal: 1.9-fold; NIC + Pal: 2.1-fold) compared to the control ethanol- and or BSA-treated groups (Figure 3(d)). We further examined total macrophage population as well as tissue-resident macrophages, which require anti-inflammatory cytokines for their maintenance in breast cancer cells treated with NIC and Pal either alone or in combination. Increased the expression of tissue-resident macrophages (CD163) expression (NIC: 3.2-fold; Pal: 1.4-fold; NIC + Pal: 3.4-fold) compared to the control group (Figure 3(d)). Since NIC has been reported to increase browning or beige adipocytes [39], which are also upregulated by anti-inflammatory cytokines, expression of beige marker TMEM26 was examined in these cells after various treatment. There was increased expression of TMEM26 in cells treated with NIC (2.4-fold) as well as NIC+ Pal (3.2-fold) treatment groups (Figure 3(d)). Since stress kinase pp38MAPK levels were upregulated in xenografts grown in the presence of HFD as well as NIC + HFD groups, we further examined pp38 MAPK in breast cancer cells following various *in vitro* treatments. There was an increase in pp38 MAPK protein expression (Pal: 2.2-fold; NIC + Pal: 3.4-fold) when compared to ethanol or BSA control groups (Figure 3(d)). Our data, therefore, show that both NIC and Pal either alone or in combination significantly increase MCSC population, anti-inflammatory cytokines, and key browning marker TMEM26 in HCC70 breast cancer cells.

### 3.4. NIC Increases MCSCs and Macrophage Population, Anti-Inflammatory Cytokines, and Adipose Browning Markers in HCC70 Cells following Incubation with Subcutaneous Adipose Tissues (SAT)

**Figure 4:**
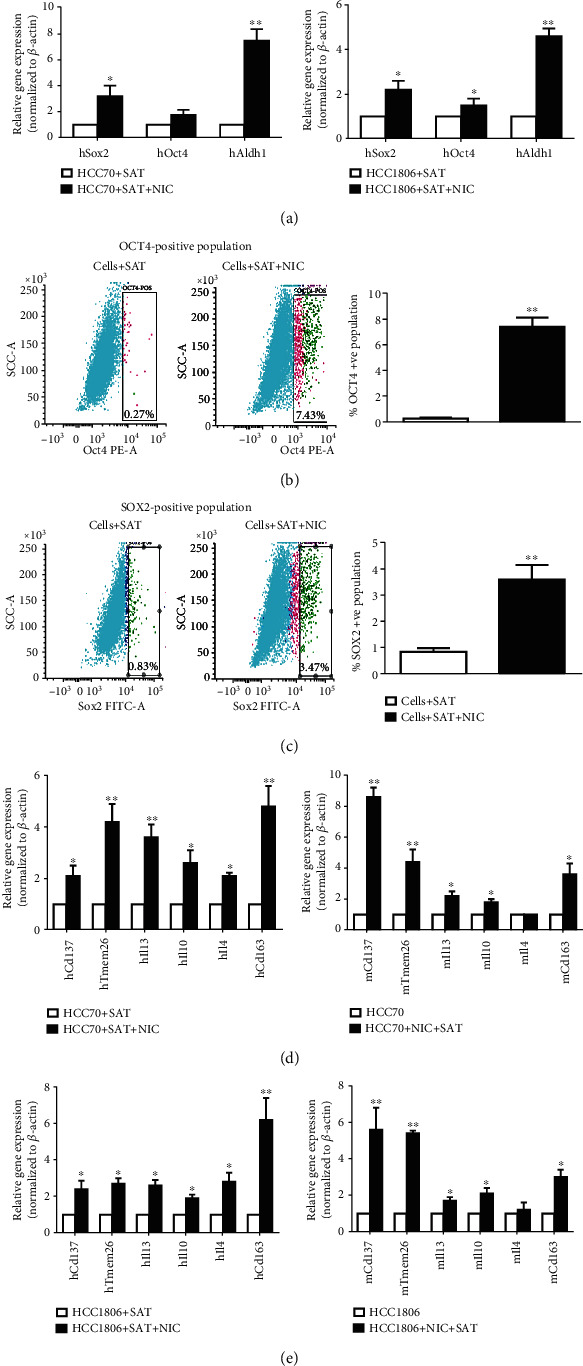
Quantitative gene expression analysis of HCC70 cells following ex vivo incubation with subcutaneous adipose tissue (SAT) from nude mice and ±NIC. HCC70 and HCC1806 breast cancer cells (5×10^6^ cells) preincubated with SAT ± NIC (1 *μ*M) for 48 h, following which (a) quantitative gene expression analysis of MCSCs was performed using human-specific primer sets: HCC70 (left panel) and HCC1806 (right panel). (b) FACS analysis showing relative changes in OCT4 population in HCC70 cells incubated with SAT and ±NIC. Representative FACS analysis (left panel) and quantitative presentation (*n* = 3) of OCT4 (right panel) is shown. (c) FACS analysis showing relative changes in SOX2 population in HCC1806 cells incubated with SAT and ±NIC. Representative FACS analysis (left panel) and quantitative presentation (*n* = 3) of SOX2 (right panel) is shown. (d, e) Quantitative gene expression analysis of HCC70 or HCC1806 with SAT + NIC for beige-adipocyte (CD137 and TMEM26), anti-inflammatory cytokines (IL13, 1L10, and IL4) and tissue-resident macrophages (CD163) using species-specific primer sets. (d) HCC70 + SAT + NIC: human (left panel) and mouse (right panel). (e) HCC1806 + SAT + NIC: human (left panel) and mouse (right panel). ^∗^*p* ≤ 0.05and^∗∗^*p* ≤ 0.01(*n* = 3).

Although we found similar patterns of expression of key tumorigenic factors during *in vivo* growth of HCC70 and HCC1806 xenografts, both cell lines were used to further investigate, the effect of NIC on adipose tissues ex vivo. We performed *ex vivo* experiments where HCC70 or HCC1806 breast cancer cells, incubated with subcutaneous adipose tissues (SAT) from naïve nude mice, were further treated with NIC. We performed qPCR after incubating both HCC70 breast cancer cells and SAT for 48 h to assess changes in MCSC, anti-inflammatory cytokines, tissue-resident macrophages, and beige adipocyte levels. There was a significant increase in gene expression of *hSox2* (3.2 ± 0.8-fold; *p* ≤ 0.05), *hOct4* (1.74 ± 0.4-fold; *p* ≤ 0.05), and *hAldh1* (7.5 ± 0.9-fold; *p* ≤ 0.01) in the cancer cells coincubated with SAT (Figure 4(a), left panel). Similar incubation of HCC1806 with SAT following NIC treatment showed significant increase in *hSox2* (2.2 ± 0.8-fold; *p* ≤ 0.05), *hOct4* (1.5 ± 0.3-fold; *p* ≤ 0.05), and *hAldh1* (4.6 ± 0.35-fold; *p* ≤ 0.01) gene expression (Figure 4(a), right panel). FACS analysis showed significantly increased levels of OCT4- (26.5-fold, *p* ≤ 0.001) in HCC70 (Figure 4(b)) and SOX2 (4.3-fold, *p* ≤ 0.01) -positive population of HCC1806 cells (Figure 4(c)) in NIC-treated breast cancer cells incubated with SAT compared to the cells plus SAT group. In these *ex vivo* experiments, human- and mouse-specific primer sets were further used to analyze expression of beige adipose (*Cd137*, *Tmem26*), tissue-resident macrophage (*Cd163*), as well as anti-inflammatory (*Il13*, *Il10*, and *Il4*) markers. Analysis of HCC70 breast cancer cells incubated with SAT and NIC showed significant increase in gene expression of *Cd137* (*hCd137*: 2.1 ± 1.0-fold, *p* ≤ 0.05; *mCd137*: 8.6 ± 0.6-fold, *p* ≤ 0.01), *Tmem26* (*hTmem26*: 4.2 ± 0.7-fold, *p* ≤ 0.01; *mTmem26:*4.3 ± 0.8-fold, *p* ≤ 0.01), *Il13* (*hIl13*: 3.6 ± 0.5-fold, *p* ≤ 0.01; *mIl13*: 2.2 ± 0.3-fold, *p* ≤ 0.05), *Il10* (*hIl10*: 2.6 ± 0.5-fold, *p* ≤ 0.05; *mIl10*: 1.8 ± 0.2-fold, *p* ≤ 0.05), *Il4* (*hIl4*: 2.1 ± 0.1-fold, *p* ≤ 0.05), and *Cd163* (*hCd163*: 4.8 ± 0.8-fold, *p* ≤ 0.01; *mCd163*: 3.6 ± 0.7-fold, *p* ≤ 0.05) (Figure 4(d)). Similar incubation of NIC-treated HCC1806 breast cancer cells with SAT led to significant increase in gene expression of *Cd137* (*hCd137*: 2.4 ± 0.46-fold, *p* ≤ 0.05; *mCd137*: 5.6 ± 1.2-fold, *p* ≤ 0.01), *Tmem26* (*hTmem26*: 2.7 ± 0.3-fold, *p* ≤ 0.05; *mTmem26:* 5.4-fold, *p* ≤ 0.01), *Il13* (*hIl13*: 2.6 ± 0.3-fold, *p* ≤ 0.05; *mIl13*: 1.7 ± 0.2-fold, *p* ≤ 0.05), *Il10* (*hIl10*: 1.9 ± 0.2-fold, *p* ≤ 0.05; *mIl10*: 2.1 ± 0.3-fold, *p* ≤ 0.05), *Il4* (*hIl4*: 2.8 ± 0.5-fold, *p* ≤ 0.05), and *Cd163* (*hCd163*: 6.2 ± 1.2-fold, *p* ≤ 0.01; *mCd163*: 3.0 ± 0.4-fold, *p* ≤ 0.05) (Figure 4(e)).

### 3.5. Treatment with nAchR Antagonist Reduces NIC plus Pal-Mediated Aggressive Breast Tumor Properties

**Figure 5:**
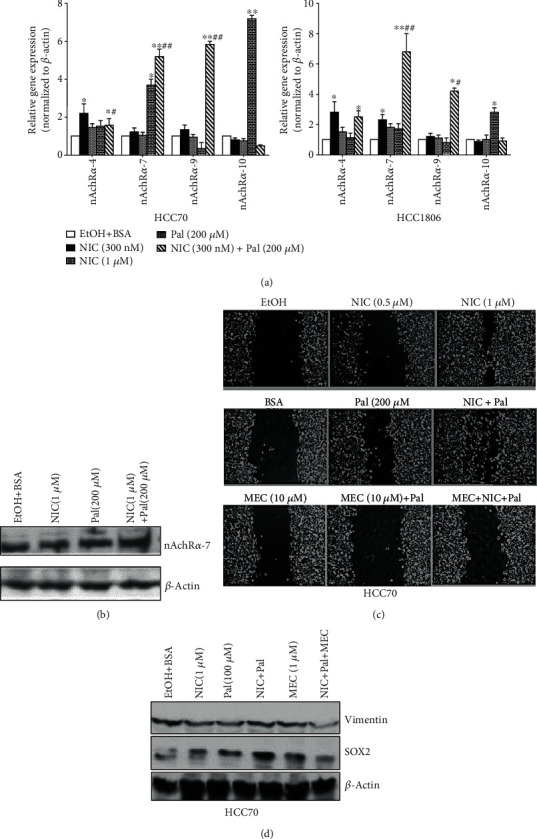
Treatment with MEC reduces NIC + Pal-mediated increase in vimentin and SOX2 in HCC70 cells. (a) Quantitative gene expression analysis of various nAchR isoforms in HCC70 (right panel) and HCC1806 (left panel) after various treatments. (b) Western blot analysis of nAchR*α*-7 in HCC70 cells treated with either NIC ± Pal for 72 h. (c) Cell invasion assay: HCC70 cells were plated in 6-well plates, grown to confluence, scratched, and further treated with NIC ± Pal with/without MEC for 72 h. (d) Immunoblot analysis of vimentin and SOX2 in HCC70 cells treated with NIC ± Pal with/without MEC for 72 hours. ^∗^*p* ≤ 0.05and^∗∗^*p* ≤ 0.01compared to saline or ^#^*p* ≤ 0.05and ^##^*p* ≤ 0.01compared to the NIC group alone (*n* = 3).

We next examined the expression of various isoforms of nicotinic acetylcholine receptors (nAchR) in HCC70 and HCC1806 breast cancer cells to determine their contribution in tumor growth in the presence of NIC and HFD. Quantitative gene expression analysis showed a significant increase in *α-7 nAchR* gene expression with 200 *μ*M Pal (3.7 ± 0.3-fold; *p* ≤ 0.05), which was further increased in the NIC + Pal (5.2 ± 0.4-fold; *p* ≤ 0.01) treatment group compared to the control group (Figure 5(a), left panel). In addition, *α-9 nAchR* and *α-10 nAchRα* gene expression was found to be significantly induced only in the NIC + Pal (5.8 ± 0.2-fold; *p* ≤ 0.01) and Pal (7.2 ± 0.2-fold; *p* ≤ 0.01) groups, respectively (Figure 5(a), left panel). Quantitative gene expression analysis of HCC1806 cells following various treatments also showed an increase in *α-7 nAchR* gene expression with 200 *μ*M Pal (1.7 ± 0.3-fold; *p* ≤ 0.05), which was further increased in the NIC + Pal (6.8 ± 1.2-fold; *p* ≤ 0.01) treatment group compared to the control group (Figure 5(a), right panel). Also, *α-9 nAchR* and *α-10 nAchRα* gene expression was found to be significantly induced only in NIC + Pal (4.2 ± 0.2-fold; *p* ≤ 0.05) and Pal (2.8 ± 0.3-fold; *p* ≤ 0.05) groups, respectively (Figure 5(a), right panel). Since HCC70 breast cancer cells lack functional ER, known to mediate oncogenic effects of NIC intracellularly [40], we examined expression levels of glucocorticoid receptors (GR) and androgen receptor (AR). We did not find any significant changes in any of the GR (GR1-3) or AR expression levels following NIC and Pal treatment alone or in combination (data not shown). Furthermore, immunoblot analysis showed no appreciable increase in the protein expression level of *α-7* nAchR with the NIC + Pal treatment (~1.16-fold) group compared to the ethanol+ BSA-treated group (Figure 5(b)). We further examined the effect of nAchR inhibition by MEC, on HCC70 cell proliferation/migration following treatment with NIC and Pal. We performed scratch-wound assay with HCC70 cells grown to confluency followed by scratches and treatments with various combinations of NIC and or Pal with or without MEC (1 *μ*M). Treatment with MEC significantly reduced NIC and Pal-induced cell proliferation/migration of HCC70 cells (Figure 5(c)). Although MEC reduced proliferation/migration, there was no change in levels of VEGF as shown by immunoblot analysis of breast cancer cells after various treatments (data not shown). On the other hand, simultaneous incubation of NIC + Pal-treated HCC70 cells with MEC resulted in decreased expression levels of vimentin (0.55-fold) and SOX2 (0.45-fold) expression compared to the NIC+ Pal-treated group (Figure 5(d)). These data indicate MEC treatment significantly attenuated NIC + Pal-mediated proliferation/migration of breast cancer cells as well as reduced levels of vimentin and SOX2 expression.

### 3.6. In Vivo Treatment with MEC and SB 203580 Inhibitors Reduced Nicotine and HFD-Induced Xenograft Growth

**Figure 6:**
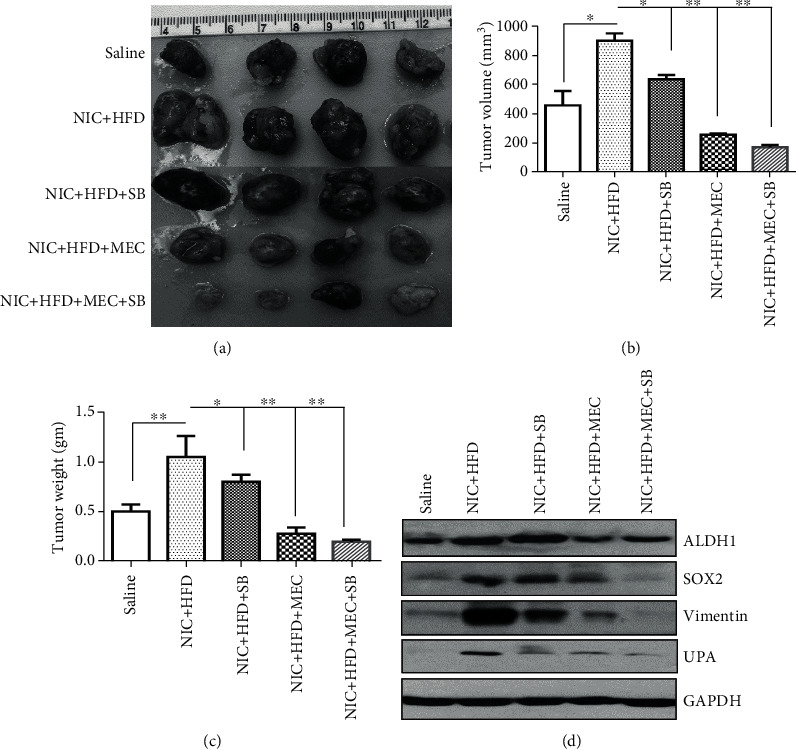
Effect of MEC and pp38MAPK inhibitor SB203580 on tumor growth in NIC and HFD-treated nude mice: HCC70 breast cancer cells (2×10^6^ cell/mice) were implanted in nude mice prefed on HFD (2 weeks) and exposed to NIC (intraperitoneal injection twice/day for 2 weeks, 0.75 mg/kg/mice/injection). Xenografts were grown in mice continued on either HFD ± NIC treatment (0.75 mg/kg/mice/injection twice a day) along with treatment with MEC (0.8 mg/kg/mice/injection, twice a day) with or without SB203580 (0.2 *μ*mols in 100 *μ*l/mice once/day/every day) till the tumors were excised. (a) Representative picture of xenografts after various treatments is shown. (b) Analysis of tumor volume following various treatments over a period of 8 weeks. (c) Analysis of tumor weight following various treatments over a period of 8 weeks. (d) Immunoblot analysis of ALDH1, SOX2, vimentin, and UPA in pooled tumors from various treatment groups (*n* = 5). ^∗^*p* ≤ 0.05 and ^∗∗^*p* ≤ 0.01.

Since xenografts exposed to HFD as well as NIC + HFD had upregulated pp38MAPK, we further examined whether inhibition of nAchR*α* and pp38 MAPK would influence xenograft development *in vivo*. Immunodeficient mice, fed HFD and exposed to IP injections of NIC (0.75 mg/kg/mice, twice a day) for two weeks, were implanted with HCC70 breast cancer cells (2×10^6^ cell/mouse). We continued the treatment of nude mice to IP injection of MEC as well as subcutaneous injection of SB 203580 in addition to HFD and NIC. The xenografts were excised after growth for another 10 weeks, after which the tumor volume and weigh were measured (Figures 6(a)–6(c)). Compared to saline (455 ± 70 mm^3^) at 10 weeks, the tumor volumes in NIC + HFD (899 ± 50 mm^3^) was significantly higher (*p* ≤ 0.05). Simultaneous treatment of the NIC + HFD group with SB (634 ± 29 mm^3^) and MEC (254 ± 18 mm^3^) alone or in combination (169 ± 16 mm^3^) resulted in significantly less tumor volume compared to the NIC plus HFD group (*p* ≤ 0.01) (Figure 6(b)). Tumor weight in NIC + HFD (1.05 ± 0.15 g) (*p* ≤ 0.01) was significantly higher compared to that in the control saline (0.5 ± 0.05 g) group (Figure 6(c)). Furthermore, cotreatment of NIC + HFD groups with either SB 203580 (0.8 ± 0.05 g; *p* ≤ 0.05) or MEC (0.28 ± 0.04 g; *p* ≤ 0.01) alone or in combination (0.19 ± 0.01 g; *p* ≤ 0.01) significantly reduced the tumor weight (Figure 6(c)). We subjected pooled tumor samples from various treatment groups to immunoblot analysis for key genes known to contribute to xenograft growth in presence of NIC + HFD. We found appreciable changes in ALDH1 (that NIC + HFD increased ALDH1 (1.65-fold) as well as SOX2 (2.1-fold) protein expression levels in xenografts, which remained high with SB 203580 treatment, but was reduced in the presence of cotreatment with MEC + SB 203580 (ALDH1: ~28%; SOX2: ~67%) (Figure 6(d)). However, NIC + HFD led to increased levels of vimentin (6.4-fold) and urokinase-type plasminogen activator (uPA) (3.6-fold), both of which are highly implicated in metastasis but were considerably reduced when cotreated with either SB (vimentin: ~52%; UPA: ~42%) or MEC alone (vimentin: ~78%; UPA: ~45%) or in combination (vimentin: ~92%; UPA: ~56%) (Figure 6(d)).

## 4. Discussion

The combination of biological and social factors contributes to health disparity and high mortality rate among AA women with breast cancer. These factors include among others poor diet, obesity, and higher exposure to risk factors including smoking [1]. There is higher frequency of AA women with TNBC that is usually of a higher stage with lymph node metastases [20]. TNBC is an aggressive subtype of breast cancer that disproportionately occurs at a high rate among younger AA women and is more likely to metastasize to the brain as compared to non-NTBC types. TNBC also has a very high rate of relapse and a poor prognosis in patients that do not achieve a complete response with neoadjuvant chemotherapy [41]. Several studies have investigated the associations between obesity/and breast cancer risk [21, 31] as well as cigarette smoking/and breast cancer risk [11, 15, 16]. However, the combined effect of the two common lifestyle risk factors, such as NIC and HFD, especially in TNBC, remains understudied. This report uses novel in vivo as well as ex vivo models to study cross-talk between the breast cancer cells and the microenvironment in the presence of nicotine and HFD. We found coexposures to both nicotine and HFD contributed to higher xenograft growth from AA TNBC cells when compared to saline, nicotine, or HFD alone. This study also elucidates some of the mechanisms that could have contributed to higher xenograft growth in the presence of NIC and HFD.

In our study, xenografts from HFD as well as NIC + HFD showed higher expression of CD68, a pan-macrophage marker, when compared to only saline or NIC. We also found higher levels of proinflammatory or Th1 cytokines, IL12 and IL6, that favor classically activated or M1macrophages in xenografts grown in HFD, when compared to the other groups. Upregulation of these markers suggested that HFD produced adipose tissue characterized dysfunctional synthesis of several adipokines and immune cell infiltration that creates a state of sustained low-grade inflammation favoring tumor growth [42]. Although M1 macrophages can increase tumor growth, high density of another subset of macrophages called tumor-associated or M2 type is associated with poor prognosis in TNBC, particularly those from AA women [43]. An anti-inflammatory microenvironment with high levels of Th2 cytokines such as IL13, IL4, and IL10 favors macrophages with M2 phenotype. Nicotine increases the anti-inflammatory microenvironment in various conditions, and our study is first to show that xenografts exposed to NIC as well as NIC + HFD expressed high levels of the anti-inflammatory cytokines, IL13 and IL10, in addition to high levels of tissue-resident or M2 macrophages. Our in *vitro* experiments demonstrated that AA TNBC cells treated with NIC and NIC + Pal expressed higher protein levels of IL13 and IL10, as well as the tissue-resident macrophage marker CD163. IHC staining of paraffin embedded xenografts from various treatment groups also showed intense clusters of IL13-expressing cells only in those tumors exposed to NIC and NIC + Pal. Interestingly, our qPCR data with validated species-specific primers shows that most of the IL6 was of host origin, while IL13 was expressed by tumor cells.

Interestingly, we also found much higher protein levels of MCSF only in xenografts developed in the presence of both NIC and HFD. MCSF, in addition to stimulating the production of several cytokines, is a crucial factor in the development and maturation of tissue-resident macrophages, which are protumorigenic [43]. Overexpression of MCSF promotes angiogenesis and other aggressive behaviors in tumors where it is also associated with poor prognosis [44]. There are reports of MCSF-promoting metastases in breast cancer where the initial growth of tumors is not affected in its absence [45]. Our scratch-wound assay data supports this assumption since we found increased proliferation/migration or faster filling up the wound in HCC70 cells treated with NIC + Pal when compared to the other controls.

Browning or beige adipocytes was also increased in NIC as well NIC + Pal-treated cells as well in *ex vivo* experiments where cancer cells were coincubated with SAT in the presence of NIC. There are reports that NIC promoted beige cells in smokers where they burn energy and help lose weight [39]. We have reported that beige adipocytes are upregulated in xenografts and patient-derived xenografts (PDX) from AA TNBC cell lines as well as patient tumors, respectively, where they contributed to tumor growth [30]. One of the mechanisms by which beige cells could increase tumor growth is by increasing reactive oxygen species (ROS), which is high in AA TN breast tumors where it promotes its growth. Beige cells secrete cytokines like NRG4 and BMP8b, which contributes to adrenergic-induced remodeling of neuro-vascular network in adipose tissue [45]. These secretions by beige/brown adipocytes promoted sympathetic axon growth as well as proangiogenic transcriptional and secretory profile that increased vascular sprouting [45].

Additionally, we present significant evidence that nicotine and HFD synergistically increase MCSCs, which are heterogeneous populations that have the potential to promote initiation and progression of breast tumors [11, 13, 21]. Others and we have reported that AATN breast cancer cells have higher ALDH1 expressing MCSCs when compared to those of other ethnic groups [35]. ALDH1 MCSCs contribute to initiation and engraftment of breast tumors as well as facilitate its interaction with the environment. In addition to ALDH1-expressing MCSCs, embryonic stem cells (ESCs) are a distinct subset of MCSCs that express embryonic transcription factors like SOX2/OCT4 and are upregulated in aggressive breast tumors [46]. In this study, we found that most ALDH1 MCSCs were of tumor cell origin with a relatively small contribution from the host. Higher protein expression of ALDH1 in xenografts exposed to NIC, HFD or coexposed to both, suggests an increase in ALDH1-MCSC population could be one of the mechanisms that influence larger growth of xenografts. We have further confirmed by FACS that treatment of breast cancer cells, *in vitro*, with NIC + Pal significantly upregulated cells that expressed ALDH1. Our *in vitro* studies where there was an increase in the number and size of mammospheres upon treatment of AA TNBC cells with NIC + Pal further suggested an increase in the MCSC population. Mammospheres propagated in low attachment plates under defined conditions enrich for MCSCs [35]. We found another subset of MCSCs, which are more embryonic become upregulated in xenografts developed in the presence of NIC as well as NIC + Pal. An increase in ESCs could be an additional mechanism that promoted larger tumor growth upon coexposure to NIC and HFD.

Stress kinase p38MAPK promotes aggressive breast tumor behaviors such as metastasis and angiogenesis [47]. Increased expression of key proteins in metastasis including urokinase plasminogen activator (UPA) and matrix metalloprotease-9 (MMP9) requires constitutive p38 alpha MAPK activity [48]. Aggressive and invasive breast tumors overexpress p38MAPK, whose increased activity has been associated with poor prognosis [49]. Treatment with SB 203580, which inhibits p38 MAPK activity, reduced proliferation and migration of highly invasive breast cancer cells, MDA-MB-231 [50]. In addition, inhibition of VEGF-C secretion in MDA-MB-231 cells by p38MAPK inhibitor, SB 203580, indicates a requirement of this kinase in angiogenesis [50]. In our study, treatment with SB 203580 alone did not have any effect on primary tumor growth in the presence of NIC plus HFD. Our data supports the findings from another study, where treatment with SB 203580 reduced metastatic potential of breast cancer cells but had no effect on its primary growth [51]. We found treatment with MEC, a nonselective nAchR antagonist, was effective in reducing the primary growth as well as decreasing the expression of vimentin and uPA in tumors developed in the presence of NIC plus HFD. However, cotreatment with both MEC plus SB 203580 was more effective in further reducing tumor volumes as well as attenuating protein expression of both SOX2 and vimentin, suggesting a casual role of both nicotine and p38 MAPK in NIC plus HFD-induced breast tumor progression. Combination therapy with both MEC plus SB 203580 may be effective in long-term suppression of NIC + HFD-exposed breast tumors as well as attenuating its metastatic potential. Understanding the signaling mechanisms that NIC + HFD upregulates in AATN breast cancer can facilitate identification of targets that may enable the development of pharmaceutical countermeasure to combat breast cancer initiation, progression, and metastasis in African American women.

Breast cancer is very heterogeneous where many key players interact and cross-talk to influence tumor growth. This study demonstrates that HFD induces inflammatory M1 macrophage-rich tumor-supporting microenvironment, which is a fertile ground for NIC to act upon to further increase additional protumorigenic factors like TAM and anti-inflammatory cytokines. While HFD and NIC individually increase various tumor-promoting factors that led to some tumor growth, their simultaneous presence and coordinated action appear to accelerate tumor growth. In addition, together, NIC and HFD upregulated important players among which was MCSF, which is a master regulator for macrophage maturation. Thus, it is apparent that the action of NIC and HFD in tumor microenvironment could be additive by bringing in various key elements together as well as synergistic by inducing novel players that contribute to tumor growth. Since both NIC and HFD had both independent and interdependent actions in a tumor microenvironment, to reduce tumor growth, we used specific inhibitors to target key mediators of NIC and HFD signaling, which was effective in reducing tumor growth.

The clinical/public health implications of our study are that a large number of women are exposed to both smoking and NRT (for the most part, available without a prescription), often for several years. While smoking leads to many cancers, including breast cancer, nicotine influences several cancers, including breast cancer. The mutagenic and tumor-promoting activities of nicotine may result from its ability to damage the genome, disrupt cellular metabolic processes, and facilitate growth and spreading of transformed cells. Our animal studies are able to understand the effects of nicotine *per se* on breast cancer proliferation. If the findings of our studies could be extrapolated to humans, the use of NRT may need to be reassessed.

## 5. Conclusion

This study demonstrates that the combination of nAchR antagonists with stress kinase inhibitors could significantly block xenograft growth in the presence of HFD plus nicotine, suggesting this combination therapy could potentially be used to treat obese breast cancer patients who smoke or use nicotine replacement therapy.

## Figures and Tables

**Table 1:** 

CD137	Human	F: TCCACCAGCAATGCAGAGTG
		R: CCAAAGCAACAGTCTTTAGAACC
	Mouse	F: AGTGGGCTGTGAGAAGGTG
		R: ACTCCGCGTTGTTGTGGTAGA

Tmem26	Human	F: ATGGAGGGACTGGTCTTCCTT
		R: CTTCACCTCGGTCACTCGC
	Mouse	F: ACCCTGTCATCCCACAGAG
		R: TGTTTGGTGGAGTCCTAAGGTC

CD163	Human	F: GCGGGAGAGTGGAAGTGAAAG
		R: GTTACAAATCACAGAGACCGCT
	Mouse	F: CAGCCGTTACTGCACACTG
		R: GTTACAATCACAGAGACCGCT

IL-4	Human	F: GCCAAGACCCTTCGAGAAAT
		R: CCGATCCTGTTATCTGCCTCC
	Mouse	F: GGTCTCAACCCCCAGCTAGT
		R: GCCGATGATCTCTCTCAAGTGAT

*β*-Actin	Human	F: CTCCTTTAATGTCACCACGAT
		R: CATGTACGTTGCTATCCAGGC

GAPDH	Mouse	F: AGGTCGTGTTGAACGGATTTG
		R: GGGGTCGTTGATGGCAACA

ALDH1A1	Human	F: GCACGCCAGACTTACCTGTC
		R: CCTCCTCAGTTGCAGGATTAAAG

ALDH1	Mouse	F: AACACAGGTTGGCAAGTTAATCA
		R: TGCGACACACAAACATTGGCCTT

IL-6	Human	F: ACTCACCTCTTCAGAACGAATTG
		R: CCATCTTTGGAAGGTTCAGGTTG
	Mouse	F: CTGCAAGAGACTTCCATCCAG
		R: AGTGGTATAGACAGGTCTGTTGG

IL-10	Human	F: GACTTTAAGGGTTACCTGGGTTG
		R: TCACATGCGCCTTGATGTCTG
	Mouse	F: CTTACTGACTGGCATGAGGATCA
		R: GCAGCTCTAGGAGCATGTGG

IL-13	Human	F: CCTCATGGCGCTTTTGTTGAC
		R: TCTGGTTCTGGGTGATGTTGA
	Mouse	F: TGAGCAACATCACACAAGACC
		R: GGCCTTGCGGTTACAGAGG

OCT4	Human	F: CAAAGCAGAAACCCTCGTGC
		R: TCTCACTCGGTTCTCGATACTG

nAchRa4	Human	F: GGAGGGCGTCCAGTACATTG
		R: GAAGATGCGGTCGATGACCA

nAchRa7	Human	F: GCTGGTCAAGAACTACAATCCC
		R: CTCATCCACGTCCATGATCTG

nAchRa9	Human	F: CAGAGACGGCAGATGGAAAAT
		R: CCACTGGACGAAGAGCATTAGAA

nAchRa10	Human	F: TCGACATGGATGAACGGAACC
		R: ATCGTAGGTAGGCATCTGTCC

SOX2	Human	F: GCCGAGTGGAAACTTTTGTCG
		R: GGCAGCGTGTACTTATCCTTCT
	Mouse	F: GCGGAGTGGAAACTTTTGTCC
		R: GGGAAGCGTGTACTTATCCTTCT

CD68	Human	F: GGAAATGCCACGGTTCATCCA
		R: TGGGGTTCAGTACAGAGATGC
	Mouse	F: TGTCTGATCTTGCTAGGACCG
		R: GAGAGTAACGGCCTTTTTGTGA

## Data Availability

All data are provided in the manuscript, and the figures are included with the manuscript.

## Abbreviations

HFD: High-fat diet
ALDH1: Aldehyde dehydrogenase 1
nAchR: Nicotine acetylcholine receptor
MEC: Mecamylamine
ER: Estrogen receptor
PR: Progesterone receptor
MCSC: Mammary cancer stem cells
TNBC: Triple negative breast cancer
AA: African American
CAA: Cancer-Associated Adipocytes
NIC: Nicotine
ATCC: American Type Culture Collection
EMT: Epithelial to mesenchymal transition
CD68: Cluster of Differentiation 68
CD163: Cluster of Differentiation 163
Pal: Palmitate
BSA: Bovine serum albumin
SAT: Subcutaneous adipose tissues
uPA: Urokinase plasminogen activator.
